# *Copernicia Prunifera* Leaf Fiber: A Promising New Reinforcement for Epoxy Composites

**DOI:** 10.3390/polym12092090

**Published:** 2020-09-14

**Authors:** Raí Felipe Pereira Junio, Lucio Fabio Cassiano Nascimento, Lucas de Mendonça Neuba, Andressa Teixeira Souza, João Victor Barbosa Moura, Fábio da Costa Garcia Filho, Sergio Neves Monteiro

**Affiliations:** 1 Department of Materials Science, Military Institute of Engineering-IME, Rio de Janeiro 22290270, Brazil; raivsjfelipe@hotmail.com (R.F.P.J.); lucio@ime.eb.br (L.F.C.N.); lucasmneuba@gmail.com (L.d.M.N.); andressa.t.souza@gmail.com (A.T.S.); 2 Science and Technology Center, Federal University of Cariri, Juazeiro do Norte 63048080, Brazil; victor.moura@ufca.edu.br; 3 Department of Mechanical and Aerospace Engineering, University of California San Diego—UCSD, La Jolla, CA 92093, USA; fdacostagarciafilho@eng.ucsd.edu

**Keywords:** *Copernicia prunifera*, carnauba fibers, epoxy matrix, natural fibers composites, characterization

## Abstract

A basic characterization of novel epoxy matrix composites incorporated with up to 40 vol% of processed leaf fibers from the *Copernicia prunifera* palm tree, known as carnauba fibers, was performed. The tensile properties for the composite reinforced with 40 vol% of carnauba fibers showed an increase (40%) in the tensile strength and (69%) for the elastic modulus. All composites presented superior elongation values in comparison to neat epoxy. Izod impact tests complemented by fibers/matrix interfacial strength evaluation by pullout test and Fourier transformed infrared (FTIR) analysis revealed for the first time a significant reinforcement effect (> 9 times) caused by the carnauba fiber to polymer matrix. Additional thermogravimetric analysis (TG/DTG) showed the onset of thermal degradation for the composites (326 ~ 306 °C), which represents a better thermal stability than the plain carnauba fiber (267 °C) but slightly lower than that of the neat epoxy (342 °C). Differential scanning calorimetry (DSC) disclosed an endothermic peak at 63 °C for the neat epoxy associated with the glass transition temperature (T_g_). DSC endothermic peaks for the composites, between 73 to 103 °C, and for the plain carnauba fibers, 107 °C, are attributed to moisture release. Dynamic mechanical analysis confirms T_g_ of 64 °C for the neat epoxy and slightly higher composite values (82–84 °C) due to the carnauba fiber interference with the epoxy macromolecular chain mobility. Both by its higher impact resistance and thermal behavior, the novel carnauba fibers epoxy composites might be considered a viable substitute for commonly used glass fiber composites.

## 1. Introduction

Sustainable action to mitigate worldwide pollution and climate changes are promoting the use of natural materials in the substitution for synthetic ones. A typical example is the use of fibers extracted from plants replacing glass fibers as reinforcement in polymer matrix composites [1,2,3]. Indeed, composites reinforcement with natural lignocellulosic fibers (NLFs) are likely to be environmentally friendly than glass fiber composites (fiberglass) in terms of biodegradability and reduced process energy [1]. Moreover, the density-rationalized specific strength of some NLF composites are superior to that of fiberglass [2]. In addition, NLFs are comparatively less expensive and nonabrasive to processing equipments, which contribute to their cost effectiveness [4]. Another relevant advantage is the social benefit since, around the world, many NLFs are cultivated in developing regions and represent a major source of income to the local population [5].

**Figure 1:**
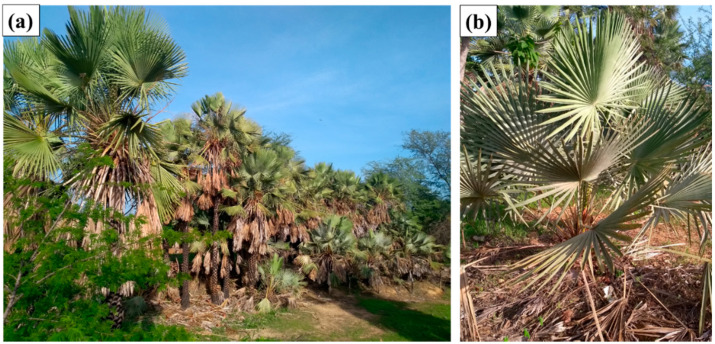
Plantation of carnaubeiras (**a**) and typical leaf-stalks of carnauba tree (**b**).

A surging in the interest for polymer composites incorporated with natural fibers in past decades motivated a significant number of research works [6,7,8,9,10,11,12,13,14,15] and industrial application [16,17,18,19,20]. In particular, less known NLFs, such as guaruman [21], buriti [22], and fique [23] have recently been successfully evaluated as reinforcement of polymer composites. Another less known NLF is the fiber extract from the leaf-stalk of the *Copernicia prunifera* palm tree, endemic in the northeastern of Brazil where it is referred to as canaubeira. Figure 1a illustrates a plantation of carnaubeiras, and Figure 1b shows their typical leaves composed of radial stalks. The main international valuable product of carnaubeira *Copernicia prunifera* is the carnauba wax worldwide commercialized in the form of yellow-brown flakes [24]. This wax has multiple applications. It can produce a glossy finish in automobile, shoes, furniture, surfboard, and floor. In addition to gloss, the hypoallergenic and emollient properties of carnauba wax justify its use in cosmetics, skin care, and even candy coating. In the US, the main importer of carnauba wax, its most common application is for paper coatings [25]. After removing the leaves, cellulose-rich stalks are considered waste and usually disposed on the soil or burnt. Sustainable destination for this increasing amount of waste could be achieved by considering its incorporation in polymer composites.

To our knowledge, a single research work on carnauba fiber incorporated polymer composites has been published so far. Melo et al. [26] investigated the 10 wt% incorporation of chemically modified short-cut carnauba fibers into biodegradable polyhydroxybutyrate (PHB) matrix composites. Mechanical properties of their untreated fibers, together with corresponding results of related composites, are presented in Table 1.

An important point to be noted in Table 1 is the relatively high tensile strength and Young’s modulus of the plain untreated carnauba fiber. It is, however, surprising that 10 wt% addition of this fiber, either untreated or chemically modified, into the PHB matrix was not able to promote any reinforcement. As such, the only published results on carnauba fiber incorporated polymer composites [26], failed to show a reinforcement effect. Based on the relatively high mechanical properties of carnauba fiber in Table 1, which are comparable to other NLFs, such as bamboo, coir, and piassava [4] used as reinforcement for polymer composites, an investigation deserves to be conducted on this less known fiber.

Therefore, in the present work the possible use of carnauba fiber as reinforcement of epoxy composites is for the first time investigated regarding the tensile properties, impact resistance by Izod tests, as well as fiber/matrix interfacial strength obtained by pullout tests. The Fourier transform infrared (FTIR) spectroscopy and thermal analysis contribute to characterize the limits for engineering applications of these composites, which are expected to be established in the future ongoing works by our research group.

## 2. Materials and Methods

### 2.1. Materials

**Figure 2:**
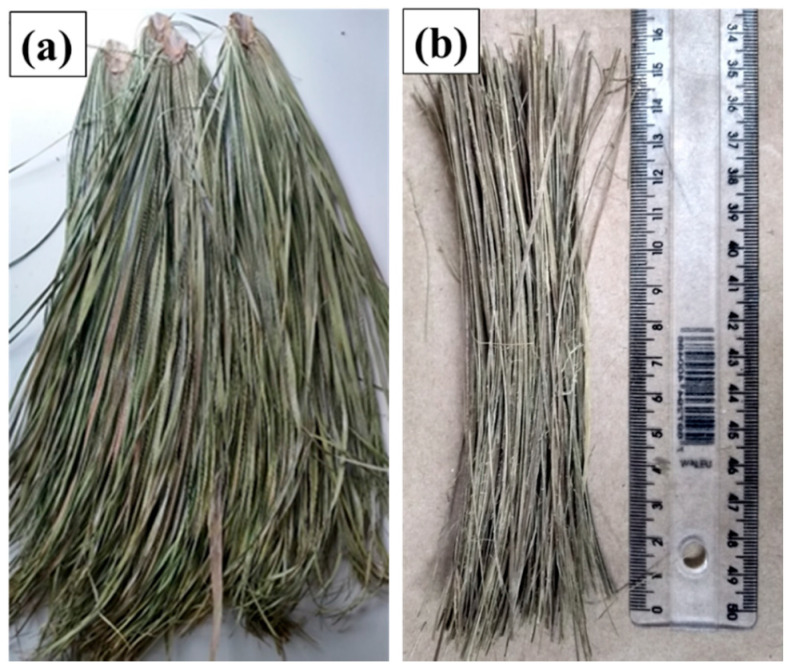
Macroscopic aspect of carnauba: (**a**) As received leaf-stalks and (**b**) shredded fibers.

Carnauba green leaves with stalks, illustrated in Figure 2a, were purchased from a rural producer in the city of Barro, state of Ceará northeast region of Brazil. The as-received leaves were subjected to immersion in water for 24 h, followed by sun-drying in open air for 12 h and then cleaning before fiber-shredding for the final aspect show in Figure 2b. Preliminary measurements of the fibers density indicate an average value of 1.13 ± 0.21 g/cm^3^.

The material used as matrix for the composite plates was a commercial epoxy resin type ether, diglycidyl bisphenol A (DGEBA), hardened with triethylene tetramine (TETA), associated with a stoichiometric ratio of 13 parts of hardener to 100 parts of resin. The resin manufacturer was Dow Chemical (São Paulo), supplied and distributed by Epoxy Fiber (Rio de Janeiro), both in Brazil.

### 2.2. Composites Processing

**Figure 3:**
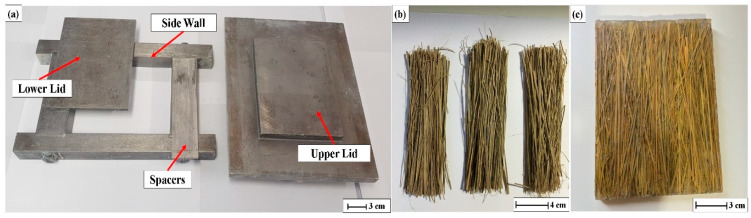
Methodology for processing the composite plates: (**a**) Metallic mold, (**b**) cleaned and dried carnauba fibers, and (**c**) cured composite plate.

The fibers shown in Figure 2b were once again cleaned, cut to 150 mm in length and finally, dried in an oven at 30 °C for 24 h, to release excess of humidity, as shown in Figure 3b. For the manufacture of composite plates, a steel mold, Figure 3a, with an internal volume of 15 × 12 × 1.19 cm^3^ was adapted to a hydraulic press (Skay, São José do Rio Preto, SP, Brazil) with maximum load capacity of 30 tons.

In order to fabricate the composite plate, continuous and aligned carnauba fibers were precisely hand lay-up along the mold’s 15 cm greater dimension. The amount of fiber for each plate corresponds to its defined volume fraction in the composite. Still fluid epoxy resin DGEBA-TETA was carefully poured into the mold avoiding fibers movement. The system was then sealed and subjected to a pressure of 5 tons for 24 h. Figure 3c shows a typical cured composite plate in which most fibers are well aligned, which confirms that fibers did not move during the 24 h pressing. For the calculation of the volume fraction of the composite, the density of 1.11 g/cm^3^ was used for the DGEBA-TETA epoxy resin [27] and 1.34 g/cm^3^ for the carnauba fibers [26]. Composite plates with 0, 10, 20, 30, and 40 vol% of fibers were prepared.

### 2.3. Pullout Test

**Figure 4:**
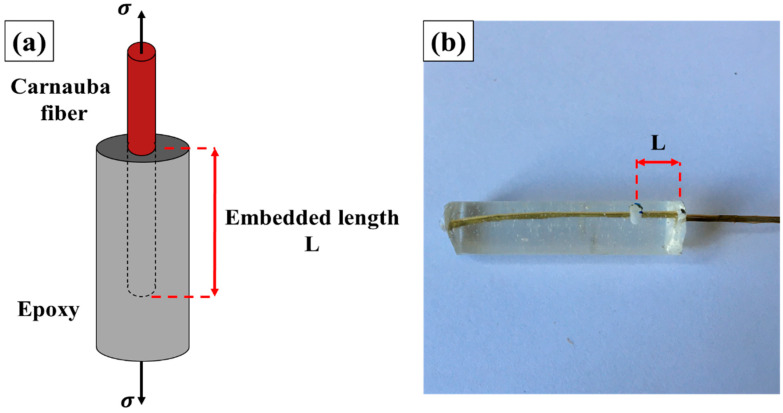
Scheme for pullout test (**a**,**b**) epoxy-carnauba specimen.

Figure 4 schematically illustrates the specimen used for the pullout test, which is composed of cylindrical blocks with 8 mm in diameter. These specimens were prepared by varying the depth of single fiber inlay embedded length of 1.5 to 30 mm, according to the methodology proposed by Kelly and Tysson [28] and adapted by Monteiro and D’Almeida [29] for NFLs. The tests were conducted at room temperature (~ 25 °C) in a model 3365 Instron universal machine (Instron Corp., Norwood, MA, USA), with a crosshead speed of 0.75 mm/min. This is a methodology which has been widely used to determine the shear strength related to the fiber/matrix interface, in particular for natural fibers [30,31,32]. After tests, specimens were gold sputtered in a vacuum desk V (Denton, TX, USA) and analyzed by scanning electron microscopy (SEM) inn a model Quanta FEG 250 FEI microscope (Field Electron and Ion Co., Hillsboro, OR, USA).

### 2.4. Tensile Test

For the tensile tests, four specimens of each group were cut from the composite plates, following the dimensions required by the ASTM D3039 standard [33], 150 × 15 × 2 mm, and gauge length of 90 mm. The tests were conducted at room temperature (~ 25 °C) in the same Instron universal machine, with a load capacity of 20 kN and a crosshead speed of 2 mm/min.

### 2.5. Izod Impact Test

Izod impact tests were carried out according to the ASTM D256-10 standard [34]. Notched prismatic specimens were machined in the direction parallel to the fiber alignment and tested using a Philpolymer instrumented pendulum model XJC 25D (Philpolymer, São Roque, SP, Brazil), using the 22 J hammer. The specimens were produced in the dimensions of 62.5 × 12.7 × 10 mm with a 2.54 mm notch transversal to the fiber alignment. A minimum of 16 specimens were tested for each volumetric fraction of carnauba fiber investigated.

The analysis of variance (ANOVA) was applied, using the F test, in order to assess whether there was a significant difference between the results obtained for Izod impact energy. The 95% confidence level was adopted for the tests, where the alternative hypothesis (H_1_) is assumed if the value of F (calculated) is higher than the critical F_c_ (tabulated), thus concluding that at the significance level of 5% there is a difference between the averages of the treatments applied. Given this information, the use of the Tukey test, known as the honestly significant difference test (HSD), becomes necessary. The objective is to quantitatively assess each of the percentages of fibers used.

The use of the Tukey test allows the comparison between the averages obtained, two by two, for each of the treatments used (percentage of fibers). From the results, it is possible to reject or not the hypothesis of equality between the averages compared through the lower significant difference (LSD), according to:

(1) $$LSD\;=q\times \sqrt{\frac{MSE}{r}}$$

By using this methodology, it was possible to quantitatively determine in a comparative way the influence of the volumetric fraction of the carnauba fibers applied in the production of the composites. The fracture surfaces of the Izod specimens together with the pullout specimens were analyzed by scanning electron microscopy (SEM) in a Quanta FEG 250 FEI microscope, operated with secondary electrons at 10 KV.

### 2.6. FTIR Analysis

The Fourier transform infrared spectroscopy (FTIR) analysis was performed in a Spectrum Two Perkin Elmer (Waltham, MA, USA) equipment. The fibers and composites were ground in the required powder condition to produce the sample tablets. The samples were scanned from 4000 to 450 cm^−1^ and the data generated were treated using the equipment data analysis program. The respective transmittance (%) spectra were generated as a function of the wave number (cm^−1^).

### 2.7. Thermal Analysis (TG/DTG)

For thermogravimetric analysis, both composites and fibers were crushed and allocated in a platinum crucible introduced in a Shimadzu model TG-50 (Shimadzu Corp., Kyoto, Japan) equipment operating with nitrogen atmosphere with a heating rate of 10 °C/min in a temperature range of 25 to 600 °C. The TG/DTG analysis followed the ASTM E1131 standard [35].

### 2.8. Differential Scanning Calorimetry (DSC)

For differential scanning calorimetry (DSC) analysis the composites and fibers were crushed and placed in an aluminum crucible. A Shimadzu model DSC-60A Plus (Shimadzu Corp., Kyoto, Japan) equipment was used. The DSC analysis was carried out under nitrogen atmosphere with a flow rate of 50 mL/min, heating rate of 10 °C/min, in the temperature range of 25 to 600 °C.

### 2.9. Dynamic Mechanical Analysis (DMA)

Dynamic mechanical analysis was carried out in order to identify important parameters such as glass transition temperature, as well as to investigate the viscoelastic behavior of composites. Specimens with 0, 10, 20, 30, and 40 vol% of carnauba fibers were fabricated according to ASTM D4065 standard [36] and the test mode was three points bending for specimens fixed by one end. The equipment used was a model DMA Q800, TA Instruments (New Castle, DE, USA), operated at a heating rate of 5 °C/min and specimens dimensions of 65 × 12 × 3 mm, from which the curves of storage modulus, loss modulus, and tangent delta were recorded.

## 3. Results and Discussion

### 3.1. Pullout Test

**Figure 5:**
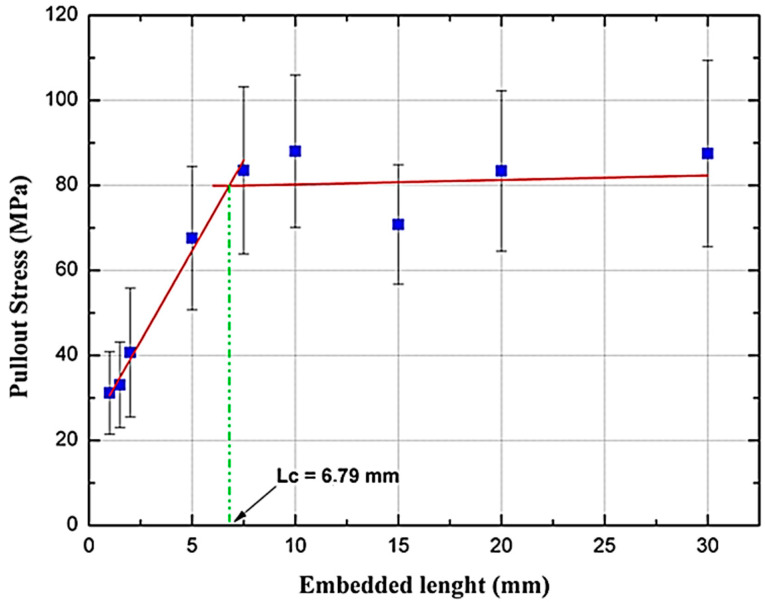
Pullout test of the different depths of carnauba fiber embedding in the epoxy resin.

Figure 5 presents the results obtained in pullout tests for the different depths of carnauba fiber embedded in the epoxy resin. The graph represents the pullout stress as a function of the fiber embedded length.

The results observed in Figure 5 follow the model proposed by Kelly and Tysson [28], being associated with two straight lines that intersect at the critical length (*L_c_*) of the carnauba fiber in relation to the epoxy matrix. The upper straight line with the lowest slope represents the linear adjustment between the maximum tensile stress values in pullout observed in the fiber embedded length range of 7.5 to 30 mm. A stress value of approximately 65 to 110 MPa, was found, which is relatively lower as compared to the range of tensile strength, 205–264 MPa, shown for the carnauba fiber in Table 1 [26]. This might be a consequence of the variability of carnauba fibers, as any NLF [4].

The linear adjustments applied to the pullout stresses of the carnauba fiber/epoxy are represented by Equation (2) with higher slope curve and Equation (3) with lower slope curve.

(2) σ=8.51 L+22.08

(3) σ=0.106 L+79.17 

It is important to note that, for embedding values lower than *L_c_* = 6.79 mm, the fibers were removed (pulled out) from the matrix, while for a higher value, an almost constant linear relation of the tensile strength with the embedded length, occurs around 65–110 MPa. In this case, when the maximum pullout stress is reached, the fiber breaks without being removed from the epoxy matrix. The interfacial resistance of the carnauba fiber in relation to the epoxy matrix was evaluated by the equation of Kelly and Tyson [28].

(4) $$\tau_{i}=\frac{d\sigma_{f}}{2L_{c}}$$

where “*d*” is the equivalent diameter of the fiber and *σ_f_* is the tensile strength of the fiber. With the value of *L_c_* = 6.79 mm it was possible to calculate the value of *τ_i_* through Equation (4), considered as the interfacial shear resistance between the fibers and the matrix [29]. The average diameter found for the carnauba fibers of 0.769 mm was used together with the average stress value of 65.17 MPa, to obtain 3.69 MPa for the carnauba fiber/epoxy matrix interfacial shear strength.

The obtained value is considered relatively low. This fact may be a consequence of the hydrophilic nature of the fibers, which contributes to the reduction of the interfacial interaction between the fiber/matrix. However, the value (*τ_i_* = 3.69 MPa) herein obtained is relatively higher than the epoxy-coconut fiber interaction (*τ_i_* = 1.42 MPa) and very close to that for the epoxy-PALF fiber interaction (*τ_i_* = 4.93 MPa) reported by Da Luz et al. [37]. The authors attributed these low values to the chemical nature of the NLFs together with the low surface roughness presented by their fibers.

**Figure 6:**
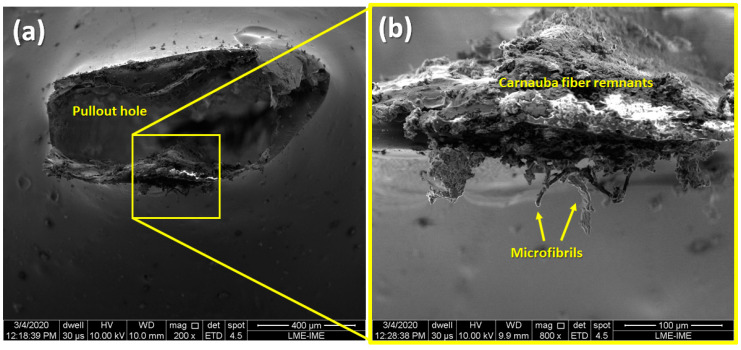
Specimens micrographs of pullout tests. (**a**) 200×, (**b**) 800×.

After the pullout test, the specimens were analyzed by SEM to obtain relevant information on the interaction between the fiber/matrix. As an example, the specimen with the embedded length of 1 mm, in which the fiber was removed by pullout from the matrix, is illustrated in Figure 6.

In Figure 6a, one clearly sees the hole left in the epoxy after the pulling out of the carnauba fiber. With the increasing magnification in Figure 6b, it is possible to note that remnants of the carnauba fiber still adhered to the epoxy matrix. These remnants can be associated with the microfibrils that were still better adhered to the matrix. In fact, after the fiber was removed, they remained attached to the epoxy resin.

### 3.2. Tensile Test

**Figure 7:**
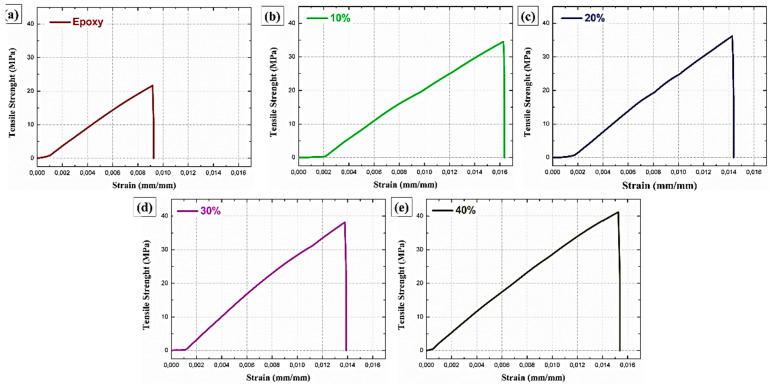
Stress-strain curves for the neat epoxy resin and composites reinforced with carnauba fibers. (**a**) Neat epoxy, (**b**) 10, (**c**) 20, (**d**) 30, and (**e**) 40 vol%.

Figure 7 shows stress-strain curves obtained in the tensile tests for the neat epoxy and the composites with 10, 20, 30, and 40 vol% of carnauba fibers.

**Figure 8:**
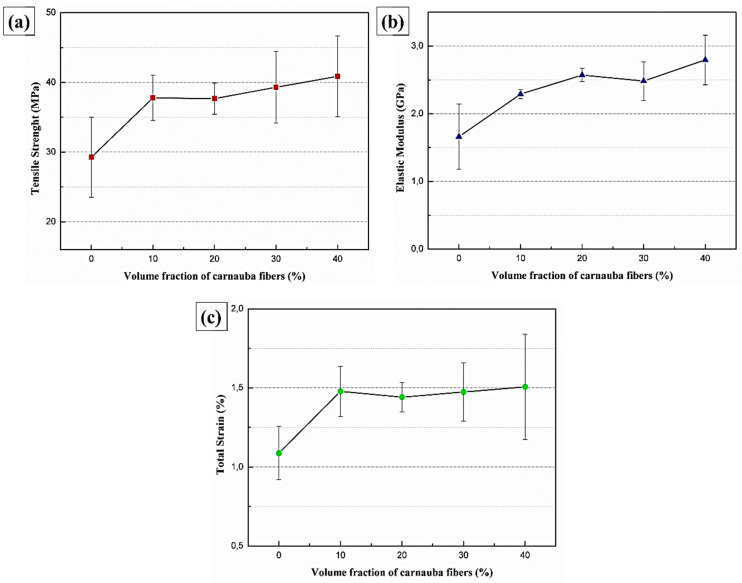
Mechanical properties as a function of the volume fraction for the neat epoxy resin and carnauba fibers reinforced epoxy composites. (**a**) Tensile strength, (**b**) elastic modulus, and (**c**) total strain.

Table 2 presents the average results for the mechanical properties for the neat epoxy and composites with different volume fractions of carnauba fibers. Figure 8 shows the graphs corresponding to the results of tensile strength, elastic modulus, and elongation presented in Table 2. The tensile strength values obtained for the DGEBA-TETA epoxy resin are comparable with values already consolidated in the literature [2,23,27]. The results show relatively superior tensile properties for composites reinforced with different volume fractions of carnauba fibers. In fact, the values of tensile strength for all composites are higher than that for the neat epoxy resin. Therefore, the carnauba fiber causes an effective reinforcement to the epoxy matrix. For instance, the 40 vol% carnauba fiber composite is 40% stronger than the neat epoxy.

According to Table 2, a tendency of increasing the composites elastic modulus with the carnauba fiber volumetric fraction is also observed. Indeed, the 40 vol% carnauba fiber composite is 69% stiffer than the neat epoxy. The composites reinforced with carnauba fibers present total strain superior (~36%) to neat epoxy resin. On the other hand, the values observed between the composites did not show any significant variation within the standard deviation.

The data presented in Table 2 highlight for the first time the reinforcement in a polymer matrix by carnauba fibers. A practical point regarding this reinforcement effect caused by carnauba fiber is the possibility of replacing a commonly used glass fiber reinforced composite in engineering applications. In fact, a comparison with DGEBA/TETA epoxy composite reinforced with 30 vol% of glass fiber [38] reveals that its specific (density-rationalized) tensile strength of 64.5 MPa.cm^3^/g is only 40% higher than that of 35.4 MPa.cm^3^/g for the 30 vol% carnauba fiber epoxy composite. By considering the aforementioned environmental and cost effectiveness advantages, the carnauba composites might be a viable substitute for fiberglass.

### 3.3. Izod Impact Test

**Figure 9:**
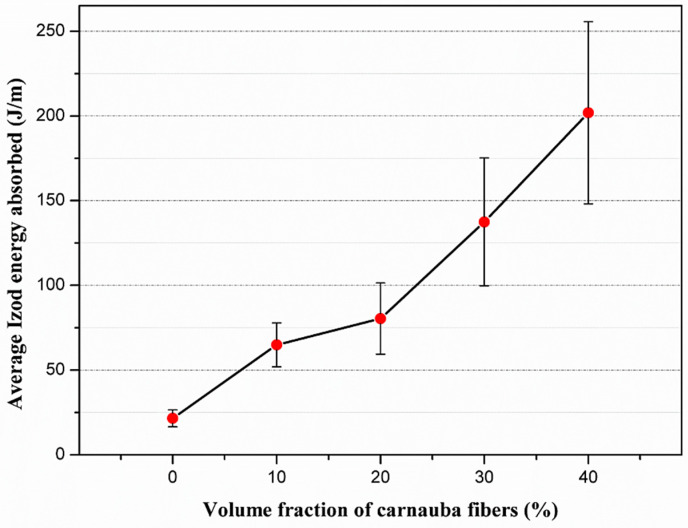
Izod impact energy as a function of the fiber fraction for the neat epoxy resin and carnauba fibers reinforced composites.

The results of the average Izod impact energy for the neat DGEBA-TETA epoxy resin and composites with different volume fractions of carnauba fibers are presented in Table 3, and graphically shown in Figure 9. The standard deviation value listed for the epoxy resin is relatively low when compared to the values associated with the composites. This fact is expected, since the NLFs present a high variability of their properties [4] and the increase of concentration in the composites, causes oscillation in their mechanical properties [38,39,40,41].

**Figure 10:**
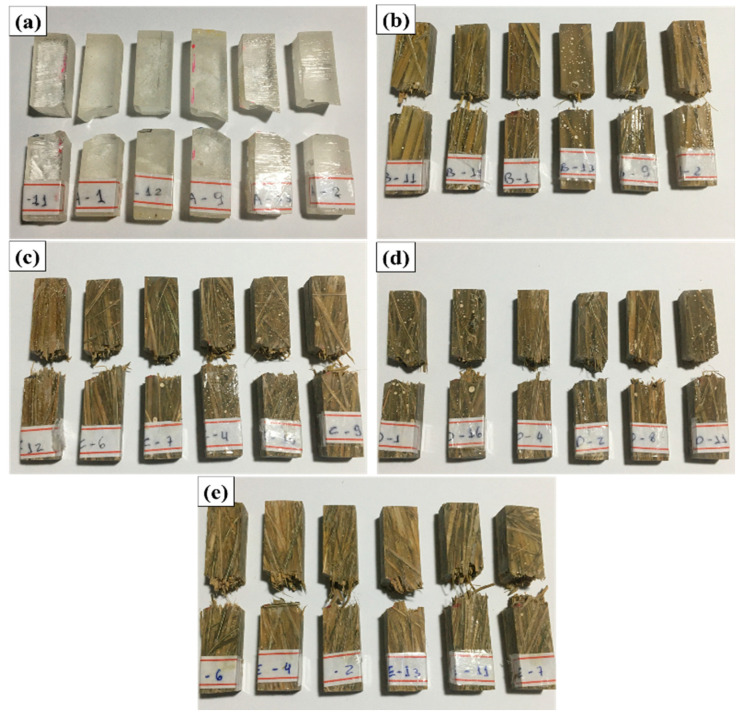
Fractured specimens after the Izod test: (**a**) 0, (**b**) 10, (**c**) 20, (**d**) 30, and (**e**) 40 vol% of carnauba fibers.

All specimens subjected to the impact test suffered a complete rupture, validating the results obtained [41]. Figure 10 shows the aspect of the broken specimens after the impact test. The visual analysis of the specimens after rupture confirms the occurrence of a fragile fracture in the specimens of pure epoxy resin, presenting a smooth and mirror surface [42]. As the volume fraction of fibers increases, it is possible to observe a greater irregularity in the fracture surface, giving evidence to the transition from fragile to ductile-fragile behavior.

With the data obtained from the ANOVA presented in Table 4, the hypothesis of equality between the averages at the 5% significance level is rejected, because F_c_ = 74.06 is higher than the F critical (tabulated) = 2.496. Therefore, the volume fraction of carnauba fibers in the composites has a direct effect on the Izod impact energy.

In order to verify which fraction of fibers presented the best results of Izod impact energy, the Tukey test was applied to compare performances with a 95% confidence level. The lower significant difference (LSD) was found as 34.04. The comparison data between the averages of Izod impact energy between the volume fractions of carnauba fibers are shown in Table 5.

Based on the results in Table 5, it was found that, with a 95% confidence level, the composite with 40 vol% of carnauba fibers, presented the best performance, exhibiting a higher average energy value of Izod impact (201.9 J/m). This proves to be a superior value in comparison with the other volume fractions of fibers, since the differences obtained are higher than the calculated LSD (34.04). It is important to note that there is no significant difference between the average Izod impact energy values of the carnauba fiber reinforcement with 10 and 20 vol%. Indeed, the values in Table 5 for the averages is not greater than the calculated LSD. This suggests that a least 30 vol% of carnauba fiber must be incorporated into the epoxy matrix for an effective reinforcement.

**Figure 11:**
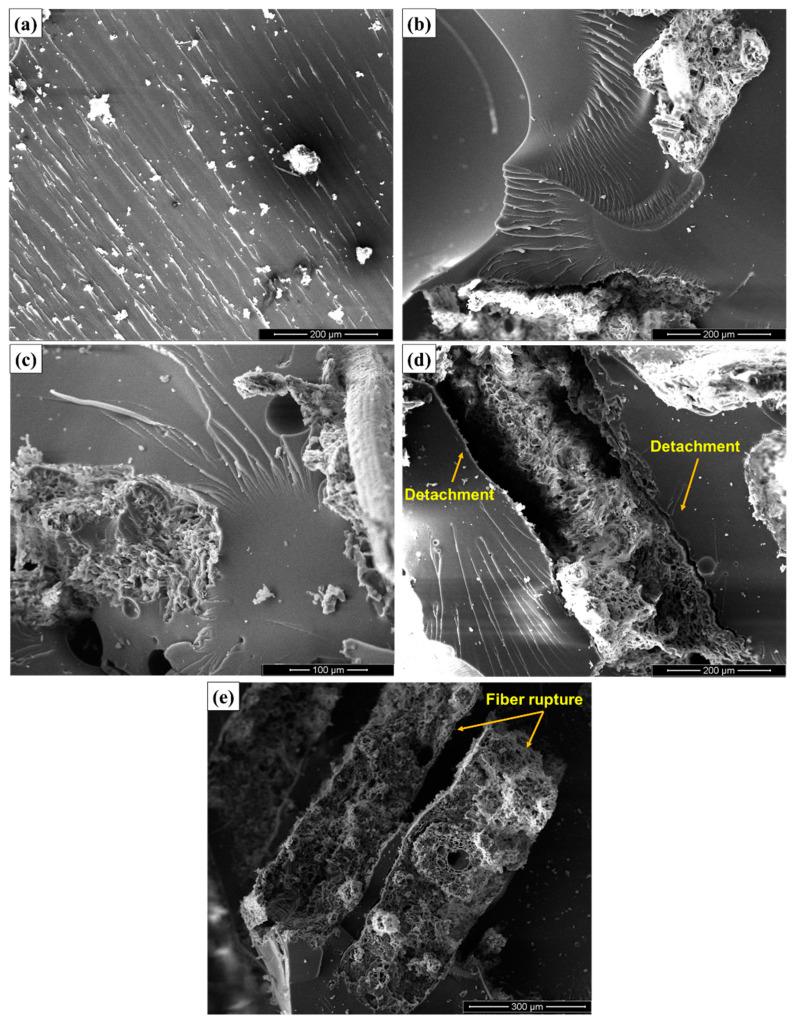
Scanning electron microscopy of fracture surfaces of composites reinforced with carnauba fibers after Izod impact tests. (**a**) 0 vol% 400×; (**b**) 10 vol% 400×; (**c**) 20 vol% 600×; (**d**) 30 vol% 400×; and (**e**) 40 vol% 300× of fibers.

The increase in impact energy with the increase amount of carnauba fibers in the composite may be related to the fracture mechanisms acting for composites of 20, 30, and 40 vol% of fibers. In order to confirm and better understand the evolution of the fracture mechanisms acting on the tested materials, SEM images of the fracture surfaces of the specimens are shown in Figure 11.

In this figure, it is possible to identify several fracture mechanisms acting on the composites. In Figure 11a, the mechanism of fragile fracture is clearly identified, due to the “river marks” present on the impact surface of the neat epoxy specimen. The same phenomenon can be observed for the composites with 10 vol% of carnauba fibers shown in Figure 11b, presenting a low effective reinforcement by the fibers for the composites with this volumetric fraction [43].

For the composites with 20 vol% of fibers, Figure 11c, the pullout of the fibers in the matrix is observed, evidenced by the circular holes shown in the fractography. For the 30 and 40 vol% composites, Figure 11d,e respectively, a more effective performance of the fibers is verified, in which the rupture and detachment of the fibers in the matrix occurred. This mechanism can be related to the absorption of high impact energies, Table 3 and Figure 9, associated with these higher volume fractions of carnauba fibers.

The incorporation of carnauba fibers in composites can be directly linked to the increase in the impact energy presented by these materials. This fact is associated with the effect of carnauba fibers as a means of interruption or deviation of the crack’s propagation direction, which contribute to a greater rupture surface area. Therefore, this causes an increase in the absorbed impact energy [27]. The important point regarding the impact results is that the carnauba fiber might indeed promote a reinforcement effect in polymer matrix composites.

### 3.4. Fourier Transform Infrared Spectroscopy (FTIR)

**Figure 12:**
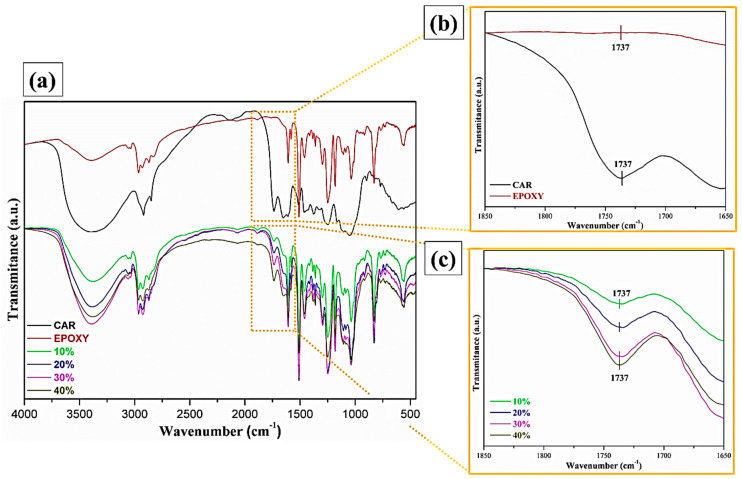
General Fourier transform infrared spectroscopy (FTIR) spectra for carnauba fiber, epoxy resin, and composites (**a**). Details for epoxy resin and carnauba fibers (**b**) and for the epoxy composites (**c**).

The FTIR spectra are presented in Figure 12, where it is possible to retrieve important information regarding the chemical structure presented by the carnauba fiber and its interaction with the epoxy resin.

The FTIR spectrum for the carnauba fiber shown in Figure 12a reveals an absorption band at 3390 cm^−1^, which is attributed to the extension of the OH bond [44]. Bands at 2890 and 2850 cm^−1^ are attributed to CH_2_ vibrations, related to molecules present in cellulose and hemicellulose [45]. The bands observed in 1653 and 1737 cm^−1^, Figure 12b, correspond to C=O bonds, probably due to functional groups (carboxylic acids, aliphatic, and ketones) present in lignin and hemicellulose [46,47]. The bands located between 1605 to 1505 cm^−1^ can be attributed to vibrations in aromatic rings [46], and in 1250 cm^−1^ refers to the stretching of the CO bonds of phenolic groups present in the fibers constituents. The alcohols present in the constitution of cellulose show vibrations of deformation associated with the OH bond and generally appear around 1360 cm^−1^ [45].

The increase in the volume fraction of fibers in the composite causes the variation in the relative absorption of the 1737 cm^−1^ band shown in Figure 12c. However, there is no significant absorption in this wavelength in the spectrum of the DGEBA-TETA resin in Figure 12b. This vibration is characteristic of C=O of functional groups present in the constituents of the fibers. As such, the proportional increase in the volume fraction of carnauba fiber in the composites is associated with an increase in the absorption of this band. One might speculate that this increase interferes with the surface adhesion between the hydrophilic carnauba fiber and the hydrophobic epoxy resin.

### 3.5. ThermoGravimetric Analysis (TG/DTG)

**Figure 13:**
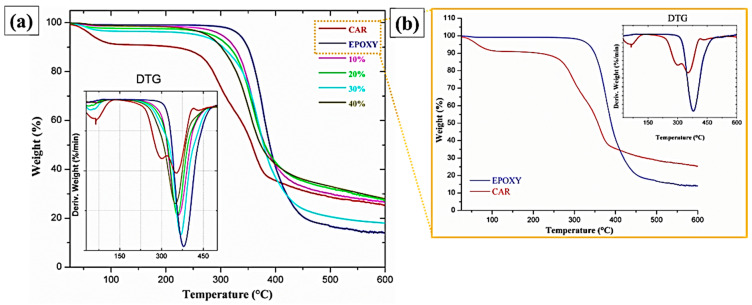
Thermogravimetric curves for carnauba fibers, resin, epoxy, and composites (**a**); carnauba fibers and epoxy resin (**b**).

As shown in Figure 13, the epoxy resin is thermally stable up to approximately 280 °C, with a negligible mass loss of 1.05%, Figure 13b. At approximately 300 °C, the greatest mass loss of the epoxy begins, at a maximum rate of 380 °C and extending up to 490 °C. This mass loss represents about 80.1% of the mass of the sample, which can be associated with the degradation of the polymer chains of the epoxy resin. The TG curves obtained for the epoxy-fiber composites with 10, 20, 30, 40 vol% fractions in comparison with the carnauba fiber and the DGEBA-TETA revealed an intermediate thermal stability in Figure 13a.

Table 6 presents the main thermogravimetric parameters, temperatures, and mass loss for the plain carnauba fiber, neat epoxy (0% fiber), and carnauba fiber composites with 10, 20, 30, 40 vol% fractions.

The results in Table 6 indicate that the composites present better thermal stability in relation to the plain carnauba fibers. Indeed, both the initial degradation (267 °C) and maximum degradation rate (353 °C) of the plain carnauba fiber are lower than the corresponding temperatures (326 °C) and (360 °C), respectively, for the 10 vol% carnauba fiber composites. However, these temperatures for all composites are slightly lower than those corresponding to the neat epoxy. Similar results were found for other less known NFLs polymer composites [48]. The composites present an average mass loss of 67% at the end of the second stage of degradation, thus showing better thermal stability than epoxy composites reinforced with a glass fiber treated with graphene oxide (GO), reduced graphene oxide (rGO), and graphene nanoplatelets (GNPs) [49]. Based on these results, a thermal stability limit of 300 °C could be considered for carnauba fiber composites.

### 3.6. Differential Scanning Calorimetry (DSC)

**Figure 14:**
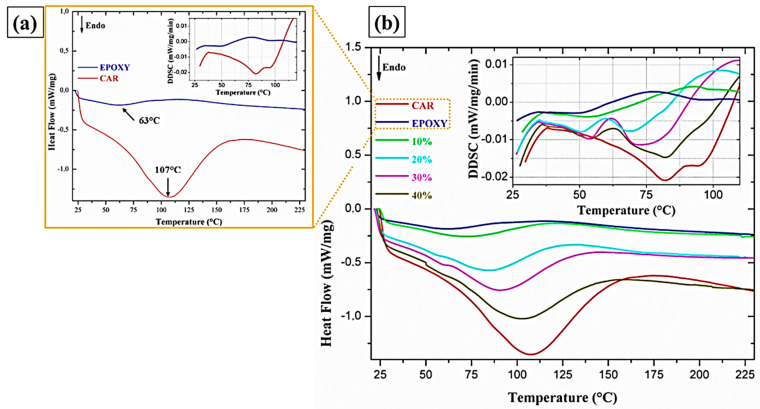
Differential scanning calorimetry (DSC) curves for epoxy resin and carnauba fibers (**a**) and (**b**) composites.

Figure 14 shows the DSC curves obtained for the epoxy resin, the carnauba fiber, and the studied composites. It is possible to observe an endothermic peak at 63 °C, associated with the glass transition temperature (T_g_) of the epoxy resin, as highlighted in Figure 14a [50]. As for the endothermic peak of the plain carnauba fiber at 107 °C, it is attributed to the release of moisture adhered to the hydrophilic surface.

The increase of the volume fraction of carnauba in the epoxy matrix causes the displacement of the endothermic peak (63 °C) observed in the DSC curve for the neat epoxy. The composites endothermic peaks in Figure 14b are probably related to both the release of moisture present in the carnauba fiber and the T_g_ of the epoxy matrix. This may explain the increase in the endothermic peaks temperature (75–103 °C) with the increase in the amount of fibers in the composite, in spite of the well-known interference of NLFs with the polymer matrix crystalline arrangement [50,51].

### 3.7. Dynamic Mechanical Analysis (DMA)

**Figure 15:**
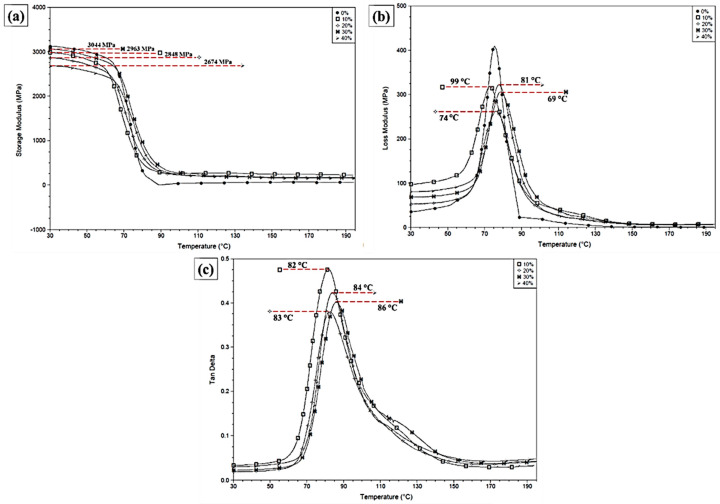
Dynamic mechanical analysis. (**a**) Storage modulus, (**b**) loss modulus, and (**c**) tangent delta for the carnauba fiber composites.

Table 7 and Figure 15 show the curves of storage modulus (E’), loss modulus (E”), and tangent delta (tan δ) of the neat epoxy [52] and different carnauba fiber composites, from 25 up to 200 °C.

The E’ results in Figure 15a reveal an improvement of the storage modulus with the incorporation of carnauba fiber, which confirms the reinforcement effect owing to its better interaction with the epoxy matrix [53]. As for the E” results in Figure 15b, the incorporation of carnauba fiber displaces the loss modulus peaks to higher temperatures, as compared to that of the neat epoxy reported elsewhere [52,54].

The E” peak is related to the structural relaxation and might be assigned to T_g_ [41]. A similar situation occurs for the tan δ peaks in Figure 15c. A comparison between the value of T_g_ for the neat epoxy obtained from DSC (63 °C) is close to that from DMA tan δ (74 °C). As for the T_g_ of the composites (82–84 °C), they are slightly higher due to the carnauba fiber interference with the mobility of epoxy macromolecular chains [52].

## 4. Summary and Conclusions

Pullout tests provided a critical embedded length of 6.79 mm for the carnauba fiber in the DGEBA/TETA epoxy resin with an interface shear strength value of 3.69 MPa, which is comparable to those presented by other NLFs.

Epoxy composites reinforced with carnauba fibers showed higher tensile strength values (37.8–40.9 MPa) than the neat epoxy (29.3 MPa), characterizing a reinforcement effect. There was also a tendency of increasing the composites elastic modulus (2.29 to 2.80 GPa) as the volume fraction increases, which was attributed to the higher stiffness of the carnauba fiber. The total strain (1.41.5%) did not show any significant variation between the different carnauba fiber composites, but is superior to the neat epoxy resin (1.1%).

Izod impact tests revealed an increase in impact energy with the volume fraction of carnauba fibers incorporated in the epoxy resin. The maximum value obtained for the Izod impact energy of 201.9 J/m, for the percentage of 40 vol% carnauba fibers is more than nine times that of the neat epoxy of 21.5 J/m. The ANOVA confirmed the highest Izod impact energy results for the epoxy composites with 40 vol% carnauba fiber and, together with the tensile properties, demonstrated for the first time the carnauba fiber reinforcement effect.

The SEM micrographs of the fracture surface revealed an evolution in the fracture mechanisms with an increase in the composite volume fraction of carnauba fibers, going from totally brittle neat epoxy (0%) to ductile-brittle for the 40 vol%. It was also possible to identify several active mechanisms.

The FTIR analysis showed expected results, with bands referring to molecular vibrations of functional groups belonging to the basic constituents of NLFs, such as cellulose, hemicellulose, and lignin. The FTIR of the composites showed a variation in the relative intensity of the 1737 cm^3^ band, thus being able to make a relationship with the intensity presented by the band and the concentration of fibers in the composite.

The TG analysis of the composites displayed an intermediate behavior on the thermal stability of the composites, when relating the thermal behavior of the neat epoxy resin and the plain carnauba fiber. The composites, on average, showed significant loss of mass above 300 °C. The increase in the volume fraction of carnauba fibers in the composite leads to a reduction in the initial degradation temperature and temperature of maximum degradation rate.

The DSC curves disclosed an increase in the temperature of the endothermic peak observed in the neat resin, with the increase in the composite volume fraction of carnauba fibers. This is mainly attributed to the moisture release from the carnauba fiber and a possible contribution to the glass transition temperature (T_g_) of the epoxy matrix.

DMA results confirm the carnauba fiber reinforcement effect associated with an improvement in the storage modulus, as compared to the neat epoxy. Moreover, the T_g_ obtained from the loss modulus and tan δ peaks, display for the composite an increase (69–99 °C) in comparison with that (64 °C) for the neat epoxy.

According to the mechanical and thermal properties presented by the carnauba fiber epoxy composites, their reinforcement effect and thermal stability above 300 °C make them viable substitutes for other epoxy composites, especially those reinforced with glass fiber.

## Figures and Tables

**Table 1:** Mechanical properties of plain untreated carnauba fiber neat polyhydroxybutyrate (PHB) and 10 wt% carnauba fiber PHB composites, reproduced from [26].

Material	Tensile Strength (MPa)	Young’s Modulus (GPa)
Neat PHB	28–30	3.3–3.6
Plain untreated carnauba fiber	205–264	8.2–9.2
10 wt% untreated with carnauba fiber/PHB composite	17–19	2.1–2.7
Maximum values for a 10 wt% chemically modified carnauba fiber/PHB composite	24–27	3.0–3.4

**Table 2:** Mechanical properties for the epoxy resin and carnauba fibers reinforced composites.

Volume Fraction (%)	Tensile Strength (MPa)	Elastic Modulus (GPa)	Total Strain (% of elongation)
0	29.3 ± 5.7	1.66 ± 0.48	1.1 ± 0.2
10	37.8 ± 3.2	2.29 ± 0.07	1.5 ± 0.1
20	37.7 ± 2.2	2.57 ± 0.10	1.4 ± 0.1
30	39.3 ± 5.1	2.48 ± 0.29	1.5 ± 0.2
40	40.9 ± 5.8	2.80 ± 0.37	1.5 ± 0.3

**Table 3:** Results of the Izod impact test for epoxy matrix composites reinforced with continuous and aligned carnauba fibers.

Fiber Fraction (vol%)	Average Izod Absorbed Energy (J/m)
0%	21.5 ± 4.9
10%	65.0 ± 13.0
20%	80.4 ± 21.0
30%	137.4 ± 37.7
40%	201.9 ± 53.8

**Table 4:** Variance analysis of average impact energies obtained for reinforced epoxy matrix composites with percentages of carnauba fibers from 0 to 40 vol%.

Variation Causes	DF	Sum of Squares	Mean Square	F(calc.)	F Critical (tab.)
**Treatments**	4	312,767.02	78,191.75	74.06	2.496
**Residue**	75	79,185.15	1,055.80		
**Total**	79	391,952.17			

**Table 5:** Results obtained for the differences (LSD) between the average values of the Izod impact energy, in the volume fractions of carnauba fibers from 0 to 30 vol%, after application of the Tukey test.

Vol.%	0	10	20	30	40
0	0	43.45	58.85	115.93	180.37
10	43.45	0	15.40	72.48	136.92
20	58.85	15.40	0	57.08	121.52
30	115.93	72.48	57.08	0	64.44
40	180.37	136.92	121.52	64.44	0

**Table 6:** Thermogravimetric parameters for the neat epoxy, plain fiber, and carnauba fiber composites.

Carnauba Fiber (vol %)	Mass Loss up to 200 °C (%)	Initial Degradation Temperature (°C)	Temperature of Maximum Degradation Rate (°C)	Mass Loss at the End of Second Stage (%)	Mass Loss at 600 °C (%)
Plain fiber	9.63	267.3	353.1	63.1	74.7
Neat epoxy	1.05	341.6	380.7	81.9	86.1
10	1.49	326.4	360.1	66.5	73.4
20	2.50	320.5	355.6	63.3	72.8
30	3.75	325.9	368.5	75.6	81.9
40	1.75	305.3	350.7	63.2	71.9

**Table 7:** Dynamic mechanical analysis (DMA) parameters.

Material	E’ (35°C)	E” T_g_ (°C)	Tan δ T_g_ (°C)	Reference
0	1352	64	72	[52,53,54]
10	2963	99	82	PW ^1^
20	2848	74	83	
30	3044	69	86	
40	2674	81	84	

^1^ PW: Present Work.
